# Anatomical mapping and quantitative functional analysis of lower limb lymphatic flow in normal cynomolgus monkeys using indocyanine green lymphography

**DOI:** 10.1371/journal.pone.0338516

**Published:** 2025-12-18

**Authors:** Jihun Kim, Gyu-Seo Bae, Yujin Kim, Jiyoung Yang, Eunsu Jeon, Jincheol Seo, Youngjeon Lee, Sungin Lee

**Affiliations:** 1 Department of Veterinary Surgery, College of Veterinary Medicine, Chungbuk National University, Cheongju, Republic of Korea; 2 National Primate Research Center, Korea Research Institute of Bioscience and Biotechnology, Cheongju, Republic of Korea; 3 Laboratory Animal Medicine, College of Veterinary Medicine, Chungnam National University, Daejeon, Republic of Korea; 4 KRIBB School of Bioscience, Korea National University of Science and Technology, Daejeon, Republic of Korea; University of Louisiana at Lafayette, UNITED STATES OF AMERICA

## Abstract

**Background:**

The lymphatic system is essential for fluid balance and immune regulation, but its anatomy and function in translational models remain insufficiently defined. Indocyanine green (ICG) near-infrared fluorescence lymphography enables real-time visualization of superficial lymphatics, yet baseline functional data in non-human primates are lacking. Cynomolgus monkeys closely resemble humans and represent a relevant model for lymphatic research. This study established baseline drainage patterns and functional contractility metrics of the lower limb lymphatic system.

**Methods:**

Five healthy female cynomolgus monkeys were studied, with a total of 10 lower limbs evaluated. Lymphatic drainage was mapped using 11 intradermal injection sites and near-infrared fluorescence imaging. For functional analysis, ICG was injected into the anteromedial and anterolateral regions, which consistently exhibited clear lymphatic channels suitable for signal extraction. Fluorescence signals were analyzed using peak–valley and wavelet methods to quantify contraction metrics. Functional parameters were assessed at multiple time points for temporal stability. Comparisons were performed within each pathway, and pooled values were used for paired comparisons between the medial and lateral pathways.

**Results:**

Anatomical mapping revealed four major drainage groups. The anteromedial, posteromedial, and anterolateral regions drained predominantly to the inguinal lymph node, whereas posterolateral regions drained mainly to the popliteal lymph node. Functionally, lymphatic contractions remained temporally stable, and medial pathways exhibited significantly higher peak frequency, wavelet mean frequency, amplitude, and wavelet amplitude compared with that of the lateral pathways, indicating more frequent and stronger contractions. Lateral pathways showed slightly greater variability in contraction rhythm.

**Conclusion:**

This study provides the first integrated anatomical and functional characterization of lower limb lymphatics in cynomolgus monkeys. The findings establish baseline parameters that may guide comparative analyses, support pathway-specific investigations, and serve as baseline data for future studies of lymphatic dysfunction and disease modeling in non-human primates.

## Introduction

The lymphatic system plays a crucial role in maintaining fluid balance, immune surveillance, and tissue homeostasis [1]. Despite its physiological importance, the lymphatic structure and function remain understudied compared to those of other organ systems [2]. This limited understanding arises in part because lymphatic vessels are anatomically difficult to identify, as they are much smaller in diameter than cutaneous veins [3]. Their walls are thin and consist of an inner endothelial lining supported by a basement membrane, surrounded by one or more layers of smooth muscle cells [4]. In addition, the lymph fluid is colorless, making these vessels invisible during surgery without staining [5]. These characteristics highlight the need for dedicated imaging approaches.

Several imaging modalities have been used to visualize the lymphatic system, including lymphoscintigraphy, magnetic resonance lymphangiography, and indocyanine green (ICG) fluorescence lymphography [6–9]. Among these methods, near-infrared fluorescence (NIRF) imaging with ICG provides high spatial resolution, real-time capability, and low invasiveness, allowing the dynamic assessment of superficial lymphatic flow without radiation exposure [10–12]. Owing to these advantages, NIRF imaging has been increasingly used in preclinical and clinical research [7–9]. Nevertheless, current applications remain limited to qualitative or semi-quantitative observations, and standardized methods for functional quantification, such as contraction frequency and amplitude, are still lacking [13].

To address this gap, recent advances in signal processing, particularly wavelet-based time–frequency analysis, have provided new opportunities to evaluate lymphatic pumping dynamics in vivo [14]. Unlike conventional peak–valley analyses, wavelet-based metrics allow temporally resolved and robust quantification of contractile activity [14]. However, most studies that have employed these methods have been limited to small animal models such as mice and rats, restricting the translational relevance of their findings to human physiology [1,13,15,16]. Therefore, translationally relevant models are required.

The cynomolgus monkey (*Macaca fascicularis*) is a valuable non-human primate (NHP) for lymphatic research because of its anatomical, physiological, and immunological similarities to humans [17–19]. A previous study successfully established a secondary lymphedema model in rhesus monkeys to simulate upper limb dysfunction after breast cancer treatment [17]. However, comprehensive baseline data on the normal anatomical architecture and physiological functions of lymphatic vessels in healthy cynomolgus monkeys remain poorly defined. Establishing such normative baseline values is essential for interpreting pathological changes and for advancing the development and evaluation of lymphatically targeted therapies in preclinical research.

This study aimed to define the anatomical drainage patterns and establish baseline values for contractile dynamics of lower limb lymphatic flow in healthy cynomolgus monkeys using ICG lymphography. These pathway-specific findings provide insights that may inform the selection of injection sites and drainage routes, guide surgical planning, and improve interpretation of lymphatic involvement in future translational and clinical research.

## Materials and methods

### Animals

Five female cynomolgus macaques (*Macaca fascicularis*), aged 8–13 years and weighing 2.77–4.06 kg, were carefully selected based on their health status as evaluated by veterinarians. All animals were specific pathogen-free and were vendor-sourced from certified external suppliers. Specifically, the macaques originated from SNBL China Biomedical (China), Orient Cam Co., Ltd. (Cambodia), and the Primate Resources Support Center (Korea). All animals were housed at the National Primate Research Center (NPRC) of the Korea Research Institute of Bioscience and Biotechnology (KRIBB). The macaques were kept under controlled environmental conditions, at a temperature of 24 ± 2 °C, relative humidity of 50 ± 5%, light intensity of 150–300 lx, and 10–20 air changes per hour, with a 12-h light/dark cycle. They were provided with a commercial primate diet (2050 Teklad Global 20% Protein Primate Diet, Harlan, Envigo, United Kingdom), supplemented with seasonal fruits, and water ad libitum. Environmental enrichment, including toys, perches, and foraging devices, was provided to encourage natural behaviors and minimize stress. When singly housed, the animals were kept in cages measuring 760 × 750 × 860 mm (W × L × H), which meet the Group 4 (<15 kg) standards specified in the Guide for the Care and Use of Laboratory Animals. In general, group housing was maintained, and animals were transferred to single cages only prior to experimental procedures, followed by an acclimatization period of 2–4 weeks during which their condition was closely monitored. Group housing facilities varied in size depending on the number of animals, for example 3150 × 1450 × 2700 mm (W × L × H) for four individuals and 4600 × 2450 × 2700 mm (W × L × H) for eleven individuals, thereby providing ample space. Even during single housing, cages were arranged face-to-face, allowing visual, auditory, and limited physical contact through wire mesh, thus maintaining social interaction. Health and welfare were monitored at least once daily by trained animal care staff; assessment criteria included general appearance, activity, appetite, hydration, social interaction, and the condition of the injection site. An annual veterinary examination was performed (physical examination, fecal analysis, and hematologic testing), and any abnormalities were promptly treated under veterinary supervision. The animals were premedicated with intramuscular ketamine (5 mg/kg) and atropine (0.05 mg/kg). General anesthesia was maintained with inhaled isoflurane at 1.5–2% in oxygen delivered via a face mask. During anesthesia, heart rate, respiratory rate, oxygen saturation, and body temperature were continuously monitored. Active warming was provided when mild hypothermia occurred. Intravenous fluids (0.9% saline) were administered to maintain hydration, and ophthalmic ointment was applied to both eyes to prevent corneal drying. No clinically relevant abnormalities were observed during anesthesia. After the procedure, the animals were observed until complete recovery in a temperature-controlled recovery area. As the procedure involved only intradermal ICG injection and non-invasive imaging, no post-procedural analgesics were administered because no pain or distress was anticipated. Humane endpoints were predefined (e.g., > 20% body-weight loss, persistent anorexia >48 h, severe lethargy or respiratory distress, self-injury, or unrelieved pain), and none were reached. No animals were euthanized, and all were returned to their home colony after study completion. All experimental procedures were reviewed and approved by the Institutional Animal Care and Use Committee of the KRIBB (permit no. KRIBB-AEC-25035).

### ICG preparation

A 25 mg vial of ICG (CellBion, Seoul, Korea) was reconstituted in 5 mL of sterile water to prepare a 5 mg/mL stock solution. This stock solution was diluted with sterile water to obtain a final working concentration of 0.625 mg/mL for injection. All solutions were prepared immediately before use and were protected from light throughout the procedure to minimize photodegradation.

### Anatomical mapping

#### Injection sites for anatomical tracing.

**Fig 1:**
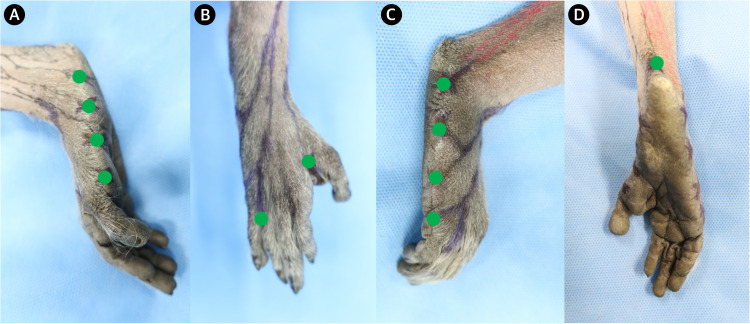
Designated intradermal injection sites in the foot. Representative photographs of the foot show the locations of indocyanine green injections (green dots). (A) Medial view. (B) Anterior view. (C) Lateral view. (D) Posterior view.

To delineate the anatomical distribution of lymphatic drainage pathways in the lower limbs, 11 intradermal injection sites were designated on each lower limb: the medial and lateral aspects of the foot (four sites each), the interdigital web spaces between digits 1–2 and 4–5 (two sites), and the plantar heel (one site). Each site received 0.1 mL of ICG at a concentration of 0.625 mg/mL using a 29-gauge needle attached to a 1 mL syringe. Injections were performed under sterile conditions with the animals placed in a supine position under general anesthesia. Care was taken to maintain consistent injection depth and volume across all sites (Fig 1).

#### Imaging conditions.

Approximately 15 min after injection, imaging was performed using a handheld NIRF camera system (FLUOBEAM^®^ FB800, Fluoptics SAS, Grenoble, France), which uses excitation light centered around 780 nm and detects fluorescence emission above 820 nm to enable real-time visualization of ICG signal. Although originally designed as a handheld device, the camera was mounted on a custom-built stabilization apparatus to minimize motion artifacts and maintain consistent imaging angles and distances across the animals and anatomical regions. The animals were maintained under general anesthesia throughout the imaging period. All the imaging procedures were performed in a light-controlled environment to optimize the signal-to-noise ratio.

#### Region definition.

To facilitate anatomical description and subsequent analysis, lymphatic drainage patterns observed during the initial mapping session were used to group anatomically adjacent injection sites into four drainage regions–anteromedial, posteromedial, anterolateral, and posterolateral. These regional definitions were applied consistently throughout the study.

### Functional assessment using ICG imaging

#### Selection of regions for functional analysis.

Among the four drainage regions identified during anatomical mapping, only the anteromedial and anterolateral pathways were included in the functional analysis. Posteromedial lymphatics, although draining toward the inguinal lymph node (ILN), coursed along the medial-posterior contour of the limb with an oblique and laterally displaced trajectory that, in the supine imaging position, resulted in depth-related attenuation, unstable fluorescence visibility, and insufficient vessel continuity for reliable time-series analysis. Posterolateral lymphatics followed a short and relatively deep pathway toward the popliteal fossa along the posterior-lateral surface, a region farthest from the optical axis in the supine position, which led to marked attenuation, limited superficial exposure, and inadequate signal continuity for reproducible contraction assessment.

#### Injection protocol.

For the functional assessment of lymphatic contractility, ICG was intradermally injected at two designated sites within the anteromedial and anterolateral regions of the hind foot, corresponding to the medial and lateral pathways, respectively. In each animal, the injections were administered unilaterally, with the medial and lateral regions of the same limb assessed in separate sessions to prevent signal overlap. ICG was first injected into the medial sites of the left lower limb, followed by a minimum 2-week washout period before injecting into the lateral sites of the same limb. Before each session, the imaging field was screened to confirm the absence of residual fluorescence. For the contralateral lower limb, the sequence was reversed, with lateral injections performed first and medial injections after washout. Each site was injected with 0.1 mL of ICG solution at a concentration of 0.625 mg/mL, using a 29-gauge needle attached to a 1 mL syringe. All injections were performed under general anesthesia with the animals placed in the supine position. No manual massage was applied post-injection to minimize the external modulation of lymphatic flow.

#### Near-infrared fluorescence imaging setup.

Functional imaging was performed using the same NIRF camera system during the anatomical mapping phase. To ensure consistent imaging conditions across animals and sessions, the device was secured on a custom-built stabilization apparatus, and the distance between the camera and imaging site was fixed at 15 cm. For each lower limb, sequential static fluorescence images were acquired at 5, 10, and 15 min post-injection. At each time point, a 3-min image series was captured with the camera oriented toward the medial thigh region to visualize the lymphatic flow toward the ILN. All imaging was conducted under general anesthesia in a light-controlled environment to optimize signal quality and minimize motion artifacts. The acquired time-series images were processed to generate video files for dynamic functional analysis.

#### ROI selection and signal processing.

**Fig 2:**
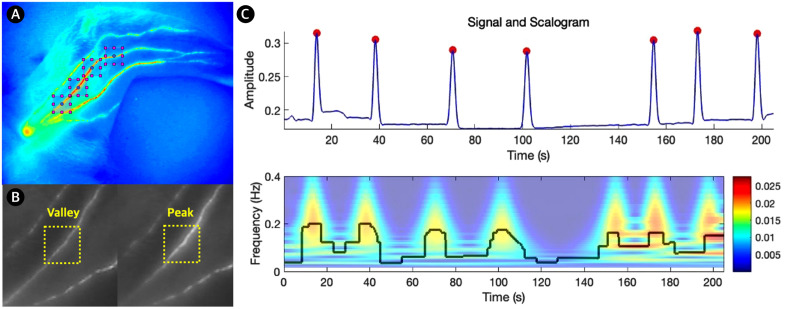
Examples of region-of-interest (ROI) selection and functional analysis of lymphatic contractility in a lower limb. (A) Near-infrared fluorescence image of inguinal lymphatic vessels, with four ROIs indicated by pink squares. (B) Example of peak and valley assignment within an ROI (yellow squares), where valleys represent periods without ICG passage and peaks represent periods of ICG transit. (C) Peak–valley intensity plot and scalogram derived from the ROI shown in (B), generated using wavelet-based time–frequency analysis to illustrate contraction frequency and temporal variability.

Image sequences acquired at 25 frames per second were analyzed using a custom MATLAB script (R2024b; MathWorks, Natick, MA, USA) kindly provided by Dr. Lance L. Munn, the corresponding author of a previously published study on lymphatic contractility quantification [14]. This code was originally developed for a multiresolution time-frequency approach combining peak–valley and wavelet-based signal decomposition. Regions of interest (ROIs) were manually selected as 50 × 50 pixel squares positioned along visible lymphatic vessels. For each injection session, four ROIs were placed on the same lymphatic drainage pathway, and the normalized intensity values (ranging from 0 to 1) were averaged across the ROIs to generate a representative signal for analysis. Signal denoising was performed using a wavelet shrinkage technique based on the Block James–Stein method, allowing optimal global and local adaptivity [20]. Two analytical strategies were then applied: (1) Peak-and-valley analysis, which estimated contraction frequency from local minima and amplitude from peak prominence; and (2) continuous wavelet transform (CWT) using the Morse mother wavelet, enabling time-resolved extraction of instantaneous frequency and amplitude. Final metrics included peak frequency, wavelet-based mean and standard deviation of frequency, and amplitude (Fig 2).

#### Quantitative metrics of lymphatic function.

Lymphatic contractility was quantified using a combination of time- and frequency-domain metrics. For time-domain analysis, the contraction frequency (expressed as peaks per minute) was calculated based on the number of local minima detected in the denoised fluorescence intensity signal. The contraction amplitude was determined from the peak prominence, representing the relative height of each peak compared with that of the adjacent troughs in normalized intensity expressed in arbitrary units (a.u.). For the frequency-domain analysis, CWT using the Morse mother wavelet was applied to extract the instantaneous frequency and amplitude at each time point. From this, the wavelet-based mean and standard deviation of instantaneous frequency (min ⁻ ¹), as well as the corresponding wavelet amplitude, were computed to assess rhythmicity and strength of lymphatic contractions. The instantaneous frequency standard deviation (IFSD) was calculated as the coefficient of variation of the instantaneous frequency, defined as the wavelet-based standard deviation of the frequency divided by the wavelet-based mean frequency, and expressed as a percentage representing the temporal variability of the contraction frequency. In addition, the mean fluorescence intensity within each ROI was measured over time to assess the ICG accumulation and baseline signal levels. Together, these parameters provided comprehensive and quantitative characterization of lymphatic function under physiological conditions.

### Statistical analysis

All statistical analyses were performed using GraphPad Prism, version 10 (GraphPad Software, San Diego, CA, USA). Functional metrics were extracted from the fluorescence intensity signals, after averaging the four regions of interest placed along the same lymphatic pathway to yield a single representative time series per session. Given the small sample size, data normality was not assumed and all analyses were performed using non-parametric tests. Within-region temporal stability was assessed separately for the medial and lateral regions using the Friedman test to compare values at 5, 10, and 15 min post-injection. The results were evaluated based on *p*-values, with statistical significance defined as *p* < 0.05. As no significant temporal effects were detected, regional comparisons were performed on the time-averaged values, calculated as the mean of the 5-, 10, and 15 min measurements for each limb and region. Paired comparisons between medial and lateral regions were performed using the Wilcoxon signed-rank test. To account for multiple comparisons across the six functional metrics, *p*-values from the Wilcoxon tests were adjusted using the Benjamini–Hochberg false discovery rate (FDR) procedure. Both unadjusted *p*-values and adjusted *q*-values are reported in the tables, with statistical significance defined as *q* < 0.05. Results are expressed as median [Q1-Q3]. No data were excluded from analysis.

### Results

### Anatomical mapping of lower limb lymphatic drainage

**Fig 3:**
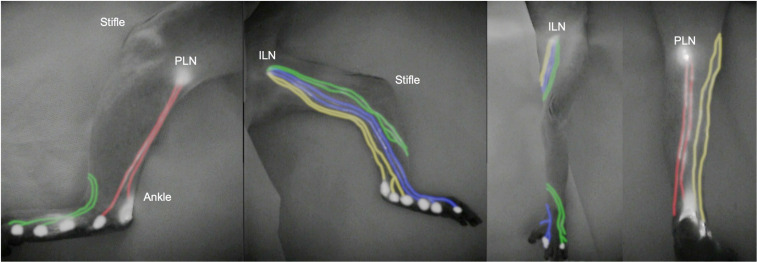
Indocyanine green fluorescence lymphography of the lower limb in a cynomolgus monkey. Lymphatic vessels are shown in a representative image and color-coded according to their regional course at the ankle: anteromedial (blue), posteromedial (yellow), anterolateral (green), and posterolateral (red). High-intensity fluorescence signals correspond to the ICG injection sites in the foot and to the inguinal lymph node (ILN) and popliteal lymph node (PLN).

**Fig 4:**
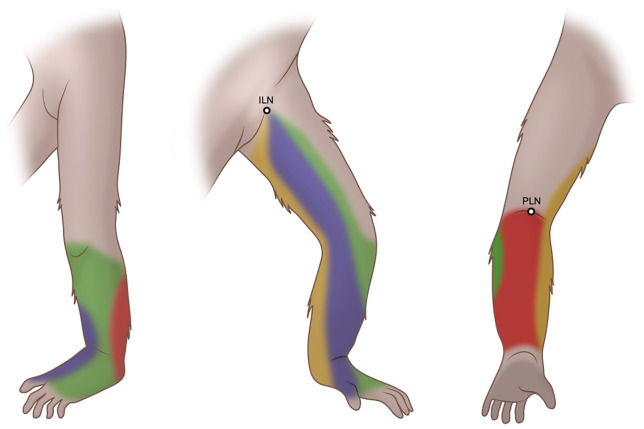
Schematic illustration of the four drainage regions in the lower limb. Anteromedial (blue), posteromedial (yellow), anterolateral (green), and posterolateral (red) regions are shown to define the regional boundaries.

In all evaluated lower limbs, NIRF imaging delineated lymphatic drainage patterns and enabled identification of pathways to the ILN or popliteal lymph node (PLN) within 15 minutes following injection. The observed drainage pathways were documented in NIRF images (Fig 3). Across all regions, lymphatic vessels generally maintained independent courses along the medial or lateral contour of the lower limb, with convergence typically occurring near the terminal lymph nodes. Most injection sites demonstrated consistent and predictable drainage patterns, although occasional variations such as vessel crossover between the medial and lateral pathways or dual drainage into multiple nodal basins were observed. Four drainage regions were defined based on these pathways (Fig 4). In all examined lower limbs, ICG fluorescence propagated along linear, unidirectional lymphatic vessels without evidence of dermal backflow, retrograde filling, or abnormal branching patterns. This finding indicates that all visualized lymphatic channels exhibited physiologic flow behavior, comparable to that observed in healthy human subjects, and that no abnormal or reflux-type drainage was detected under the present experimental conditions.

#### Anteromedial region.

Injections into the first interdigital web space and the anteromedial foot consistently produced lymphatic vessels ascending along the medial contour of the lower limb toward the ILN. In all ten lower limbs, the medial interdigital and anteromedial foot injections consistently drained into the ILN. At the ankle level, two to four vessels are typically visible from medial injections, with most retaining their separation up to the inguinal region. When convergence occurred, it was usually between adjacent anteromedial sites at the mid- to proximal lower limb, producing a single vessel proximally. The fusion of lateral-derived vessels was rare.

#### Posteromedial region.

Lymphatic vessels from the posteromedial foot generally coursed proximally toward the ILN. At the ankle level, one to two vessels were typically visible, and in several limbs, the posteromedial vessels curved medially in the proximal lower limb before merging with the anteromedial pathways toward the ILN. No direct anatomical connections with the laterally derived vessels were identified in this region. In one of the 10 limbs (10%), direct drainage to the PLN was observed, in which the posteromedial vessel entered the popliteal fossa in parallel with the posteriorly derived vessels.

#### Anterolateral region.

Injections into the fourth interdigital web space and anterolateral foot consistently produced lymphatic vessels ascending along the lateral contour of the lower limb before crossing medially, most often at or just below the knee, in the proximal portion of the lower limb. Less commonly, the medial crossover occurred near the ankle. These vessels typically continued independently toward the ILN without merging with the medial-derived vessels until after the crossover point. In all ten lower limbs, the anterolateral and lateral interdigital injections consistently drained into the ILN. At the ankle level, 1–4 vessels were typically visible, and in some cases, multiple lateral vessels converged proximally into two main trunks before entering the inguinal region.

#### Posterolateral region.

Lymphatic drainage from the posterolateral foot, including the heel, was primarily directed toward the PLN. All heel injections consistently drained into the PLN, although dual drainage was observed at two sites, with one vessel coursing medially toward the ILN and the other entering the PLN. Popliteal entry typically occurred via one or two vessels arising from the heel or the adjacent posterior regions. Among the posterolateral foot injection sites, eight of 10 limbs (80%) drained directly to the PLN, whereas the remaining 2 of 10 limbs (20%) branched proximally and connected to the anterolateral group before reaching the ILN. At the foot level, posterolateral-derived vessels were generally observed as one or two distinct vessels.

### Functional evaluation of superficial lymphatic contractility

#### Time-dependent variation in lymphatic function.

No statistically significant temporal changes were detected at 5, 10, and 15 min post-injection in either the medial or lateral region, as determined by Friedman tests. In the medial region, the peak frequency (*p* = 0.316), wavelet mean frequency (*p* = 0.187), IFSD (*p* = 0.601), amplitude (*p* = 0.368), wavelet amplitude (*p* = 0.135), and mean intensity (*p* = 0.436) remained stable across time points. Similarly, in the lateral region, no significant differences were observed in the peak frequency (*p* = 0.135), wavelet mean frequency (*p* = 0.974), IFSD (*p* = 0.948), amplitude (*p* = 0.187), wavelet amplitude (*p* = 0.974), or mean intensity (*p* = 0.187). No outliers or systematic time-dependent shifts were observed, and the range of values at each time point showed a substantial overlap across the limbs. These findings confirmed that lymphatic contractile activity remained temporally stable during the 15-min observation period in both regions, allowing reliable pooling of data across time points.

#### Regional differences in lymphatic contractility.

**Fig 5:**
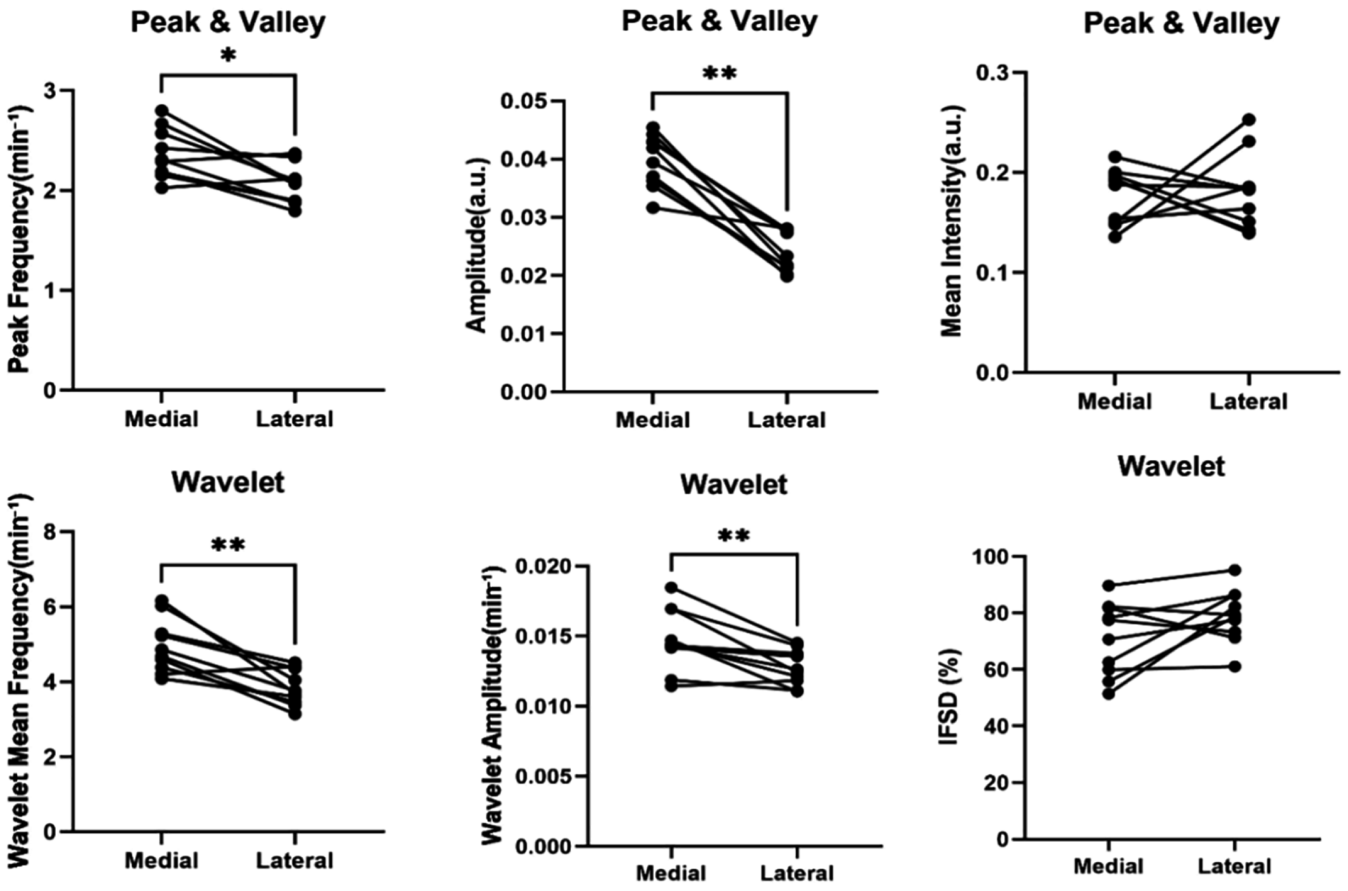
Paired comparison of lymphatic contractility metrics between medial and lateral pathways. Peak–valley analysis demonstrated significantly higher peak frequency and amplitude in the medial pathway compared with that of the lateral pathway, with no difference in mean intensity. Wavelet-based analysis further revealed higher mean frequency and amplitude in the medial pathway, whereas instantaneous frequency standard deviation did not differ significantly. These regional differences were consistently observed across individual limbs, as visualized in the paired line plots. Each line represents paired data from the same limb. *q < 0.05, **q < 0.01 (Wilcoxon signed-rank test with Benjamini–Hochberg correction).

Paired comparisons between the medial and lateral regions revealed significant functional differences for several parameters (Table 1, Fig 5). Peak frequency was ~ 11% higher in the medial pathway than in the lateral pathway (2.30 vs 2.07 min ⁻ ¹; *q* = 0.021), and wavelet mean frequency was ~ 28% higher (4.77 vs 3.73 min ⁻ ¹; *q* = 0.008), indicating more frequent contractions. Contraction strength, reflected by amplitude (approximately 78% higher) and wavelet amplitude (approximately 18% higher), was also markedly greater in the medial pathway. In contrast, the mean fluorescence intensity did not differ significantly between the regions (*q* = 0.922), suggesting comparable ICG accumulation. Although not significant, the IFSD tended to be higher in the lateral pathway (*q* = 0.157), implying a slightly greater variability in the contraction rhythm. Collectively, the data demonstrate a robust functional distinction between the medial and lateral pathways, supported by convergent evidence from both time-domain and frequency-domain analyses.

**Table 1:** Functional lymphatic contractility metrics in medial and lateral pathways.

	Medial (Median [Q1–Q3])	Lateral (Median [Q1–Q3])	*p*-value	*q*-value
Peak Frequency (min^-1^)	2.30 [2.17–2.60]	2.07 [1.89–2.17]	0.014	0.021
Wavelet Mean Frequency (min^-1^)	4.77 [4.34–5.47]	3.73 [3.44–4.37]	0.004	0.008
Amplitude (a.u.)	0.0406 [0.0361–0.0434]	0.0226 [0.0211–0.0278]	0.002	0.008
Wavelet Amplitude (a.u.)	0.0148 [0.0136–0.0170]	0.0125 [0.0117–0.0139]	0.004	0.008
Mean Intensity (a.u.)	0.190 [0.149–0.200]	0.183 [0.149–0.197]	0.922	0.922
Instantaneous Frequency Standard Deviation (%)	74.13 [58.90–81.90]	78.96 [72.87–86.35]	0.131	0.157

Median values with interquartile ranges [Q1–Q3] are shown for peak frequency, wavelet mean frequency, amplitude, wavelet amplitude, mean intensity, and instantaneous frequency standard deviation. Comparisons between medial and lateral pathways were performed using the Wilcoxon signed-rank test. Both raw *p*-values and Benjamini–Hochberg false discovery rate-adjusted *q*-values are presented.

## Discussion

To the best of our knowledge, this study provides the first comprehensive characterization of the anatomical drainage patterns and functional contractility metrics of the lower limb lymphatic system in healthy cynomolgus monkeys using ICG lymphography. Anatomical mapping revealed distinct inguinal and popliteal drainage territories, with medial and interdigital sites directed toward the ILN and posterior sites preferentially directed toward the PLN. Functionally, the medial pathway exhibited significantly more frequent and stronger contractions than the lateral pathway, as reflected by the higher peak frequency, wavelet mean frequency, amplitude, and wavelet amplitude. No significant temporal variation was observed in contractility metrics at 5, 10, and 15 min post injection. These findings establish baseline values for superficial lymphatic function in the NHP model and provide a framework for interpreting pathological changes in future preclinical studies.

Lymphosomes, which are anatomically defined skin territories that consistently drain into specific nodal basins, provide a useful framework for interpreting regional lymphatic architecture [3]. Our in vivo mapping in cynomolgus monkeys revealed territory-specific drainage patterns, broadly consistent with these known facts. Specifically, the anteromedial, posteromedial, and anterolateral regions predominantly drained into the ILN, paralleling the human superficial lymphatic system, in which lymphatics typically follow the course of the great saphenous vein and terminate in the ILN [6,21]. Meanwhile, the posterolateral region predominantly drained into the PLN, resembling the human anatomical pattern where the lymphatics accompany the short saphenous vein before entering the PLN [3]. Therefore, the organizational logic of lymphosomes is largely conserved in NHPs.

The posterior drainage pathways merit particular attention because of their clinical implications. In humans, the posterolateral territory has been identified as the principal source of popliteal lymphatic drainage, and popliteal node metastases have been documented in distal lower-limb melanoma and selected soft tissue sarcomas, such as synovial sarcoma [22,23]. Our findings in cynomolgus monkeys, showing consistent heel drainage and predominant posterolateral flow to the PLN, parallel these reports and emphasize that popliteal routes represent a reproducible component of the lower-limb lymphatic anatomy. A careful consideration of these pathways in future translational studies may be important for avoiding potential underestimation of metastatic spread or incomplete assessment of lymphatic function.

This study highlights the methodological value of using multiple, anatomically distributed injection sites to evaluate lymphatic pathways. Conventional lymphoscintigraphy protocols in humans often rely on a single interdigital injection in the first web space of the foot, or occasionally, the dorsum of the foot [24–26]. While this approach is generally sufficient to visualize inguinal-directed pathways, our data indicate that single interdigital injections may underrepresent posterior popliteal routes, suggesting the need for multiple injection sites to achieve comprehensive mapping. By systematically including the medial, lateral, interdigital, and heel sites, we delineated four distinct drainage territories and identified both inguinal and popliteal pathways. This approach has also been validated in human cadaveric and imaging studies [6]. This strategy provides a more complete anatomical framework, captures clinically relevant variability, and provides direct sentinel node mapping. From an oncological perspective, tumors arising from different sites on the foot may drain into distinct nodal basins; neglecting such regional specificity could result in false-negative sentinel node evaluations or incomplete staging [21].

To determine whether lymphatic contractility remained stable over time following ICG arrival, we evaluated multiple post injection time points. No significant time-dependent changes in any contractility metric were detected at 5, 10, and 15 min after ICG arrival in either the medial or lateral pathways. This stability indicates that once the dye reaches the lymphatic vessels, the intrinsic pumping activity rapidly enters a steady operational state and remains stable under controlled physiological conditions. Time required for ICG to reach the lymphatic collectors can vary among individuals, which may affect early measurements [27]. Therefore, a stabilization period is recommended prior to analysis. Accordingly, we restricted the analysis to data acquired 5 min after ICG arrival to improve the reliability of the functional assessment. The key point from these findings is that functional analysis could be performed at any time point within the 5–15 min window, without altering the interpretation, thereby providing flexibility in experimental design and ensuring comparability across subjects and studies.

In this study, the medial pathway exhibited a higher peak frequency, wavelet mean frequency, amplitude and wavelet amplitude, indicating stronger and more regular lymphatic contractions than the lateral pathway, which showed a slightly greater IFSD, suggestive of temporal irregularity. These differences cannot be considered evidence of functional superiority but rather as a reflection of inherent anatomical and physiological distinctions between the two routes. The medial lymphatic pathway, which typically courses along the inner limb toward the ILN, follows a shorter, straighter trajectory with fewer anatomical obstructions. In contrast, the lateral pathway traverses a longer and more circuitous route, potentially involving more vessel curvature, nodal junctions, and areas susceptible to mechanical compression near the lateral ankle, which may influence contractile function, as indicated by previous studies demonstrating regional differences in lymphatic pumping behavior across anatomical locations [28–30]. Although direct quantification of branching was not performed, lateral-flowing lymphatics frequently demonstrated multiple visible channels merging or diverging at the mid-limb levels, implying greater anatomical complexity.

Such structural complexity may impair the synchronized contraction of lymphangions, which are segmentally arranged contractile units located between the lymphatic valves that are responsible for unidirectional lymph propulsion [29]. Per previous studies, lymphangion pumping behavior is sensitive to vessel geometry, external forces, and transmural pressure, all of which can vary with anatomical configuration [30,31]. Therefore, disruption of lymphangion coordination could explain the higher variability in contraction intervals observed in the lateral pathway. These findings highlight the importance of considering regional anatomy when interpreting lymphatic function and emphasize the value of pathway-specific baseline data in translational studies on lymphatic dysfunction.

Wavelet-based time–frequency analysis has been applied to overcome the limitations of conventional peak–valley methods for quantifying dynamic lymphatic contraction behavior [14,32]. Previous assessments of lymphatic function have primarily relied on transit time measurements or peak–valley analysis, which provide a limited quantitative evaluation of lymphatic contraction [26]. Peak–valley analysis assumes signal stationarity and is less robust to noise, waveform irregularities, or gradual frequency shifts [14,33]. In contrast, the wavelet framework preserves the temporal localization of the frequency content, enabling simultaneous assessment of the dominant contraction rate, beat-to-beat variability, and time-dependent spectral shifts [14,34,35]. This approach allowed a more comprehensive and robust characterization of spatiotemporal contractile patterns in lymphatic vessels. In the present analysis, increased temporal irregularity in the lateral pathway was captured more clearly, supporting the functional interpretation of mediolateral differences that may otherwise be obscured.

This study has some limitations. The small sample size may have limited the ability to capture inter-individual variability and precluded subgroup analyses by sex, age, or body size. NIRF imaging is limited to superficial lymphatic vessels, making it difficult to evaluate deeper connections such as those between the ILN and PLN. In addition, nonspecific uptake and signal saturation of ICG may affect the accuracy of absolute flow quantification. Although cynomolgus monkeys are a relevant NHP model, species-specific differences should be considered when extrapolating to humans. Future studies with larger and more diverse populations, including pathological models, will help improve the generalizability of the results. Despite these limitations, the present findings establish baseline functional parameters of primate lymphatic contractility that may provide a foundation for future translational studies, including sentinel lymph node mapping and investigations of lymphatic dysfunction, such as lymphedema.

## Conclusion

By integrating detailed anatomical mapping with quantitative functional assessment, this study provides the first pathway-specific baseline values for the superficial lower-limb lymphatic system in healthy NHPs. The medial and lateral pathways exhibit distinct normal ranges of contractility metrics, serving as a baseline for future comparative studies and pathway-specific analyses. These findings may inform selection of injection sites and drainage pathways, surgical planning, and interpreting patterns of lymphatic involvement. The standardized analytical protocol also has potential for broader application across research settings and species.

## Supporting information

S1 Table Raw data of lymphatic contractility metrics. Excel spreadsheet containing individual values for all animals and limbs (medial and lateral pathways).
(XLSX)

S2 Video Medial pathway lymphatic contraction. (MP4)

S3 Video Lateral pathway lymphatic contraction. (MP4)
